# Evolution of a horizontally acquired legume gene, albumin 1, in the parasitic plant *Phelipanche aegyptiaca* and related species

**DOI:** 10.1186/1471-2148-13-48

**Published:** 2013-02-20

**Authors:** Yeting Zhang, Monica Fernandez-Aparicio, Eric K Wafula, Malay Das, Yuannian Jiao, Norman J Wickett, Loren A Honaas, Paula E Ralph, Martin F Wojciechowski, Michael P Timko, John I Yoder, James H Westwood, Claude W dePamphilis

**Affiliations:** 1 Intercollege Graduate Program in Genetics, Institute of Molecular Evolutionary Genetics, Penn State University, University Park, PA 16802, USA; 2 Huck Institutes of the Life Science, Penn State University, University Park, PA 16802, USA; 3 Department of Plant Pathology, Physiology and Weed Science, Virginia Tech, Blacksburg, VA, 24061, USA; 4 Department of Plant Breeding, Institute for Sustainable Agriculture, IAS-CSIC, Córdoba, 14080, Spain; 5 Department of Biology and Institute of Molecular Evolutionary Genetics, Penn State University, University Park, 16802, PAU.S.A; 6 Chicago Botanic Garden, Glencoe, IL, 60022, U.S.A; 7 School of Life Sciences, Arizona State University, Tempe, AZ 85287-4501, USA; 8 Department of Biology, University of Virginia, Charlottesville, VA 22904, U.S.A; 9 Department of Plant Sciences, University of California, Davis, CA 95616, U.S.A

**Keywords:** Parasitic plants, Horizontal gene transfer, *Phelipanche*, *Orobanche*, Legume, KNOTTIN, Albumin 1, Evolution

## Abstract

**Background:**

Parasitic plants, represented by several thousand species of angiosperms, use modified structures known as haustoria to tap into photosynthetic host plants and extract nutrients and water. As a result of their direct plant-plant connections with their host plant, parasitic plants have special opportunities for horizontal gene transfer, the nonsexual transmission of genetic material across species boundaries. There is increasing evidence that parasitic plants have served as recipients and donors of horizontal gene transfer (HGT), but the long-term impacts of eukaryotic HGT in parasitic plants are largely unknown.

**Results:**

Here we show that a gene encoding albumin 1 KNOTTIN-like protein, closely related to the albumin 1 genes only known from papilionoid legumes, where they serve dual roles as food storage and insect toxin, was found in *Phelipanche aegyptiaca* and related parasitic species of family Orobanchaceae, and was likely acquired by a *Phelipanche* ancestor via HGT from a legume host based on phylogenetic analyses. The KNOTTINs are well known for their unique “disulfide through disulfide knot” structure and have been extensively studied in various contexts, including drug design. Genomic sequences from nine related parasite species were obtained, and 3D protein structure simulation tests and evolutionary constraint analyses were performed. The parasite gene we identified here retains the intron structure, six highly conserved cysteine residues necessary to form a KNOTTIN protein, and displays levels of purifying selection like those seen in legumes. The albumin 1 xenogene has evolved through >150 speciation events over ca. 16 million years, forming a small family of differentially expressed genes that may confer novel functions in the parasites. Moreover, further data show that a distantly related parasitic plant, *Cuscuta*, obtained two copies of albumin 1 KNOTTIN-like genes from legumes through a separate HGT event, suggesting that legume KNOTTIN structures have been repeatedly co-opted by parasitic plants.

**Conclusions:**

The HGT-derived albumins in *Phelipanche* represent a novel example of how plants can acquire genes from other plants via HGT that then go on to duplicate, evolve, and retain the specialized features required to perform a unique host-derived function.

## Background

Horizontal gene transfer (HGT) is the nonsexual transmission of genetic material across species boundaries [1,2]. HGT is well known in bacteria, where HGT often results in adaptive gains of novel genes and traits [3-5]. There are fewer well-documented cases of HGT among eukaryotes [6] and the large majority of these cases appear to result in short-lived, nonfunctional sequences [6-8]. Consequently, the long-term evolutionary impact of HGT in multicellular eukaryotes remains largely unknown. Several cases of HGT are known or suspected in plants [9-23], most involving mitochondrial sequences, and/or parasitic plants [13-15,17-20,23-25]. Parasitic plants form direct haustorial connections with their host plants and are capable of obtaining a wide range of macromolecules from their hosts, including viruses [26], gene silencing signals [27], and messenger RNAs [28]. Consequently, parasites may have many opportunities for HGT events and an increased likelihood that some of these result in functional, and potentially adaptive, gene transfers. Two recent reports by Yoshida et al [19] and Xi et al [25] were the first indications that nuclear protein coding sequences, likely obtained from their respective host species, could be integrated into the genomes of parasitic plants by HGT. These were important advances, but they provided few clues as to the long term impact of HGT, how the transgenes evolve, and how they may function. We hypothesized that systematic analysis of genome-scale datasets from parasitic plants could lead to evidence for acquisition and long-term maintenance of functional gene sequences in plants that had been acquired via HGT.

Albumin 1 genes are known only from a subset of species in the legume family (Leguminosae) of angiosperms where they encode seed storage proteins and insect toxins [29,30]. The albumin 1 proteins in legumes are 112 to 154 amino acids in length and rich in cysteine residues. They form a unique protein structure known as a KNOTTIN, which has three disulfide bonds and is characterized by a “disulfide through disulfide knot” [31]. The KNOTTINs are famous for their intruguing “disulfide through disulfide knot” structure and have been extensively studied in various fields, most of which are related with potentials in drug design [32-37]. Albumin 1 genes may have originated early in the diversification of papilionoid legumes [29,30]**,** but multiple homologous gene copies have been found only in species that are members of the more derived “Millettioid s.l.” and “Hologalegina” clades [38].

*Orobanche s. l.,* often known by the common name “broomrape,” includes 150-170 obligate parasitic plant species in the family Orobanchaceae. Growing evidence supports the segregation of broomrapes into four genera [39]: *Aphyllon* (syn. *Orobanche* sect. Gymnocaulis), *Myzorrhiza* (syn. *Orobanche* sect. M.), *Phelipanche* (syn. *Orobanche* sect. *Trionychon*), *Orobanche* s. str. (syn. *Orobanche* sect. O.). Most broomrape species have a narrow host spectrum and grow exclusively on perennial eudicot host plants [40], with members of the Leguminosae, Solanaceae, and Asteraceae among the more common hosts [41]. As a member of order Lamiales, Orobanchaceae is phylogenetically well-separated from host members in these lineages, particularly legume hosts in the rosid order Fabales (Additional file 1: Figure S1; [42]). A few broomrape species (e.g., *P. aegyptiaca, P. ramosa*, *O. cernua*, *O. crenata*, and *O. minor)* have become devastating pests of important crop plants, affecting their growth and resource allocation and imparting significant losses in yield [43]. *P. aegyptiaca*, the focal species in this study, has a broad host range that includes members of the eudicot families Apiaceae, Asteraceae, Brassicaceae, Cucurbitaceae, Leguminosae, and Solanaceae.

Here we show that a gene encoding albumin 1 KNOTTIN-like protein, closely related to the albumin 1 genes, only known from papilionoid legumes, serving dual roles in food storage and as insect toxins, was found in *Phelipanche aegyptiaca* and related parasitic species of family Orobanchaceae, and was likely acquired by a *Phelipanche* ancestor via HGT from a legume host based on phylogenetic analyses. According to genomic sequences from nine related parasite species, 3D protein structure simulation tests, and evolutionary constraint analyses, the broomrape xenogene we identified here retains the intron structure, six highly conserved cysteine residues necessary to form a KNOTTIN protein, and displays levels of purifying selection like those seen in legumes. The albumin 1 xenogene has evolved through >150 speciation events over ca. 16 million years, forming a small family of differentially expressed genes that may confer novel functions in the parasites.

## Results

The albumin 1 transcript was first identified as a HGT candidate in the transcriptome of *P. aegyptiaca* (cultured and grown on Arabidopsis and tobacco) using a BLAST-based [44] bioinformatic screen (details in Material and Methods). Albumin 1 transcripts were then searched further, using BLASTX, against the NCBI nr database and the PlantGDB database [45]. Top hits were seen (Additional file 2: Figure S2) to *Medicago truncatula* albumin 1 sequences, with expected values of 5e-51 and 1e-48. Additional BLAST, including Hidden Markov Model (HMM)-based psi-BLAST searches with the sequence from *P. aegyptiaca* were performed to attempt to detect homologs in three other members of Orobanchaceae with large transcriptome datasets (two parasites, *Striga hermonthica* and *Triphysaria versicolor*, and the nonparasitic *Lindenbergia philippensis*[46]) (Parasitic Plant Genome Project, PPGP [47]). Several large public databases, including Phytozome [48], PlantGDB, and SOL Genomics Network [49], were also searched. After searching 34 sequenced genomes and transcriptomes of 274 additional plant species, albumin 1 homologs were detected only in legumes and the transcriptome libraries of *P. aegyptiaca*.

**Figure 1:**
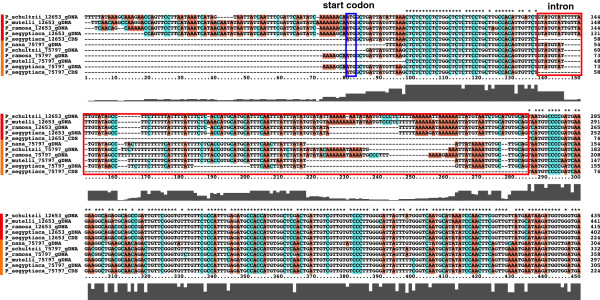
**Alignments of 5’ ends of the genomic and inferred CDS sequences of albumin 1 homologs from five *Phelipanche *species (for 3’ end, see Additional file****4****: Figure S4).** Two genes are identified from *P. aegyptiaca* unigene 12653 (first five sequences, red bar) and unigene 75797 (yellow bar)*.* Red box indicates the intron region identified by comparison of the genomic DNA and cDNA sequences. Blue box indicates the putative translation start codon.

Having identified the albumin 1 sequence in the *P. aegyptiaca* transcriptome, genomic sequences encoding albumin 1 were then obtained from *P. aegyptiaca* and eight additional *broomrape* species, including *P. schultzii, P. ramosa, P. mutelli, P. nana, and Orobanche hederae, O. minor, O. cernua and O. ballotae*. The nucleotide sequence and inferred gene structures of the albumin 1 genes in broomrape species (Figure 1; Additional file 3: Figure S3, Additional file 4: Figure S4, Additional file 5: Figure S5) are closely comparable, with inferred protein alignments 57.3-58.3% identical and 72.7%-74.3% similar (= identity + conservative substitutions) in ungapped regions between the legume and parasite proteins. Two albumin 1 genes were identified in *Phelipanche* species, and are identified here as copy_12653 and copy_75797, or albumin1-1 and albumin1-2, respectively. An intron disrupts the coding region at the same position in both genes and the intron sequences are similar but contain a number of insertion and deletion mutations. Only one albumin 1 gene was detected from *Orobanche* species. Although the intron length in albumin 1 genes of *Phelipanche* and legume species is not well conserved, several critical intron features are shared (Additional file 5: Figure S5). First, the starting position of the intron in both the *P. aegyptiaca* and *M. truncatula* sequences are the same, and the first nine base pairs are identical. Second, the introns have characteristic splicing sites at their 5’ and 3’ ends; 5’ ends often have GT/GU and 3’ ends often have AG, and these motifs are found in both *M. truncatula* and *Phelipanche* albumin 1 introns (Figure 1 and Additional file 5: Figure S5). Albumin1 gene sequences from *Phelipanche* were also searched with BLASTn against the NCBI nt database in order to search for high frequency repeats and mobile elements, but no such features were identified.

**Figure 2:**
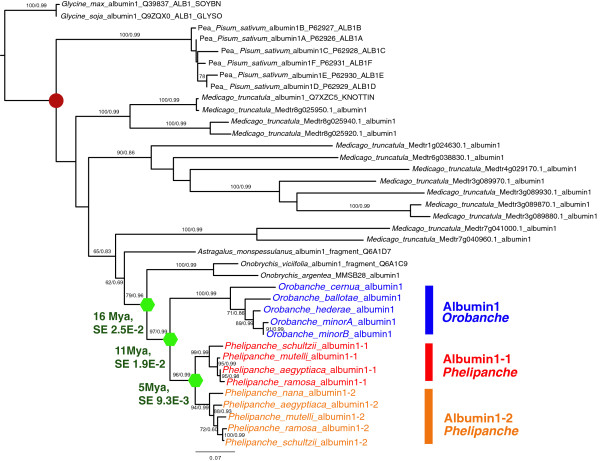
**Maximum likelihood (ML) and Bayesian inference (BI) phylogeny of albumin 1 homologs in broomrape species and legumes.** Horizontal acquisition of albumin 1 by an ancestral *Phelipanche*/*Orobanche* species was estimated to have occurred ca. 16 million years ago (Mya, with standard errors SE), with Orobanche-Phelipanche speciation ca. 11 Mya, and a gene duplication ca. 5 Mya in the *Phelipanche* lineage produced xenparalogous genes designated Albumin1-1 (12653) and Albumin1-2 (75797) (see Supplemental Methods). Papilionoid legumes in black, others as indicated. Age estimate of legume node marked by red circle (39 ± 2.4 Mya) taken from Lavin et al. [51]. Unrooted trees have been rooted with *Glycine max*, in agreement with a prior KNOTTIN phylogeny [30] and phylogenetic relationships of related legume sequences [50]. Tree shown is ML (BI method produced the same tree topology); bootstrap values (if >50%) and posterior probabilities (if >0.60) are shown on internal nodes. The legume clade containing albumin 1 genes is comprised of the Millettioids clade, which contains genera such as *Glycine* and *Phaseolus*, as the sister group to the large, temperate Hologalegina clade, which includes *Medicago*, *Pisum*, *Astragalus* and *Onobrychis*, as well as several other agriculturally important genera such as *Cicer*, *Lens*, *Vicia*, and *Trifolium*[50]. Legume KNOTTIN sequences were from the KNOTTIN database [31]. For each legume KNOTTIN, tripartite names are given as: species full name-ID from KNOTTIN database-sequence ID from UniProt database. Additional albumin 1 homologs from *M. truncatula* were retrieved from Medicago truncatula HapMap Project [52] with original sequence IDs. Branches are scaled by number of substitutions. The two albumin 1 genes in *Phelipanche aegyptiaca* have nt sequence identity 92%.

Phylogenetic analysis (Figure 2) of all known plant albumin 1 sequences showed a strongly supported clade containing all of the albumin 1 sequences from broomrapes (Maximum likelihood (ML) boostrap 98, Bayesian inference (BI) Posterior probabilities (PP) 0.99) nested deeply within the IRLC (Inverted Repeat-lacking clade) of papilionoid legumes [50]. Among legumes, the next most closely related sequences (ML bootstrap 100, BI PP 0.99) are from *Onobrychis argentea* and *Onobrychis viciifolia*. Because the node supporting the position of the broomrape clade (ML bootstrap 79, BI PP 0.99) within the papilionoid legumes is relatively weakly supported, we also tested the hypothesis that the broomrape clade of albumin 1 sequences falls outside the larger clade of legumes represented in this analysis (i.e., at a position sister to the Millettioid and Hologalegina clades). This hypothesis was rejected (Shimodaira-Hasegawa test and Kishino-Hasegawa test, using Tree-Puzzle version 5.2, Log L = -4482.60) relative to the maximum likelihood position as indicated in this tree. Two albumin 1 genes are resolved as sister clades in *Phelipanche* species, which are in turn resolved as sister to the single gene obtained from *Orobanche* species. Gene structures supported a similar conclusion (Figure 1).

**Figure 3:**
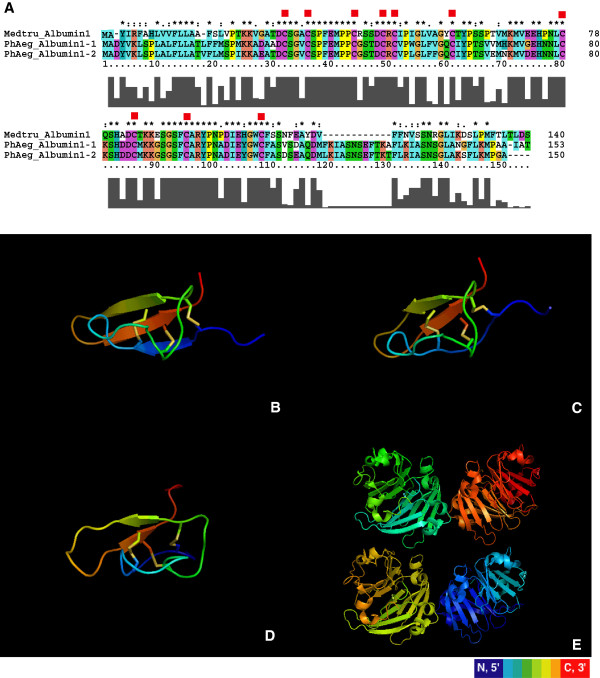
**Amino acid sequence alignment and 3D structure simulation of albumin 1 sequences from *Medicago *and *P. aegyptiaca*.** (**A**) Amino acid alignment for the two *P. aegyptiaca* albumin 1 sequences and a *M. truncatula* albumin 1 sequence (Q7XZC5, a confirmed KNOTTIN insect toxin protein). Red squares indicate cysteine residues. (**B**) and (**C**) show the simulated 3D structures for both *Phelipanche* sequences. Protein 2D structures are colored from N-terminal to C-terminal with a rainbow color scheme. The three disulfide bonds are shown as colored sticks. The left most and right most sticks open a space that is pierced by the stick in the center. This “disulfide through disulfide knot” is the characteristic structure of KNOTTIN proteins. (**D**) 3D structure of the KNOTTIN insect toxin protein in *M. truncatula*. The toxicity of this protein to insect herbivores was confirmed in an earlier report [29]. The PDB file for this 3D structure was obtained from the KNOTTIN database. (**E**) Predicted albumin 2 (a non-KNOTTIN albumin, PDB ID#3LP9) protein 3D structure in grass pea (*Lathyrus sativus*).

The amino acid sequence alignments of albumin 1 from legumes to *P. aegyptiaca* show conservation of all cysteine residues essential for disulfide bond formation in albumin 1 proteins (Figure 3A). We investigated whether the predicted albumin 1 proteins from parasites maintain the characteristic KNOTTIN structures found in the legume albumin 1 proteins using Knoter1d [31,53]. Simulated 3D structures show that the *Phelipanche* albumin 1 proteins form a characteristic KNOTTIN structure with three-disulfide bonds and a “disulfide through disulfide knot”. KNOTTIN protein structures are also predicted in all of the other full-length albumin 1 genes in *Phelipanche* species. Knoter1d assigned scores greater than 35 to each *Phelipanche* albumin 1 sequence; a score greater than 20 in this analysis passes the Knoter1d criteria for identification as an albumin 1 structure. The predicted 3D structures for *P_aegyptiaca*_Albumin1-1 (Figure 3B) and *P_aegyptiaca*_Albumin1-2 (Figure 3C) are very similar to the insect toxic albumin 1 protein from *M. truncatula*. Albumin 2, a non-KNOTTIN legume protein, has no discernable homology with the albumin 1 protein in legumes (Figure 3E).

**Figure 4:**
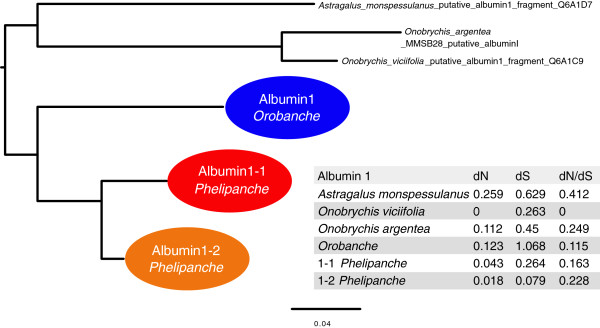
**ML estimate of dN and dS changes, and evolutionary constraint (dN/dS) through the history of albumin 1 sequences in broomrapes and their homologs in three related legume species.** Branch lengths scaled by total number of substitutions. Because the total amount of evolutionary change on individual branches for closely related species can be very low (or even zero in some cases), changes have been pooled within several of the specific lineages.

Having found that the horizontally acquired albumin1 genes were present in related species of broomrapes we then asked if the genes are evolving under purifying selection indicative of a functional protein coding sequence. dN (nonsynonymous substitutions), dS (synonymous substitutions) and dN/dS were calculated for all three lineages of the broomrape albumin 1 clade (= albumin 1 in *Orobanche*, albumin1-1 and albumin1-2 in *Phelipanche*) and for the albumin 1 sequences from three closely related legume sequences; *Astragalus monspessulanus, Onobrychis argentea* and *Onobrychis viciifolia*. Synonymous substitutions in the albumin 1 genes (dS) outnumber non-synonymous substitutions (dN) by at least 3:1 in most lineages (Figure 4), and dN/dS, reflecting the level of purifying selection, is similar in broomrapes to the value estimated for closely related albumin1 sequences from legumes. All cysteine residues were also identified as evolving under purifying selection, suggesting that the horizontally acquired albumin 1 genes in broomrapes are functional (Bayes factors ranging from 3.04 to 27.52.)

**Figure 5:**
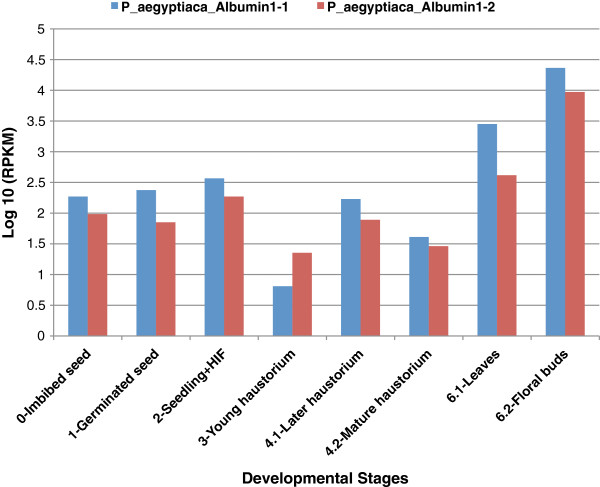
**Expression level (log scale) of *P. aegyptiaca *albumin 1 genes in *P. aegyptiaca *across eight developmental stages.** Normalized expression levels were estimated by RPKM (= count of mapped Reads to this gene Per Kilobase of sequence length per Million library reads). Numerical values in Additional file 6: Table S1; *P. aegyptiaca* stages are as defined [54] and in Additional file 7: Table S2. Stage 3 (haustorium attached to host root, pre-vascular connection) is the earliest post-attachment stage for this parasite [54].

Having observed evidence for selection for structural conservation, we investigated whether these genes exhibit transcription profiles that suggest a new or unique pattern of expression in parasites. Normalized expression levels of both albumin 1 genes in *P. aegyptiaca* were estimated as reads per kilobase per million reads (RPKM) for eight libraries representing major stages of belowground and aboveground parasite development (Figure 5, Additional file 6: Table S1, Additional file 7: Table S2). Both genes displayed lowest expression levels at stage 3 (haustorial attachment stage) and highest at stage 6 (above-ground tissues). Transcripts were particularly abundant at stage 6.2 (reproductive), more than 1000x higher than the haustorial stage.

## Discussion

Biogeographic overlap and common feeding interactions between diverse broomrapes and temperate papilionoid legumes increase the likelihood that the HGT event occurred in a common ancestor of the parasites that was in direct contact with legume host plants. An alternative (and less parsimonious) explanation is that another organism or virus that co-occurred in the same habitats as the ancestral lineages served as a “stepping stone” for a two- or more step transfer. However, this is not supported in our searches of the sequence databases. Based on fossil-calibrated age estimates of legume lineages [50,51], we estimate that this horizontal acquisition occurred in an ancestral broomrape that lived in the Miocene epoch, about 16 Mya. Both parasite and their legume host groups have northern temperate distributions, and their lineages likely overlapped in the past as they do now, providing a minimal requirement for a horizontal gene transfer to occur. Another possibility, however unlikely, is that albumin1 was a more recent acquisition that underwent strong convergence at the protein level with this legume lineage. However the branch lengths we observed in the phylogeny (Figure 2) were not unusually long in our DNA-based phylogeny, and given the large collection of related sequences we obtained from other broomrape species, we have reduced any tendency the *Orobanche/Phelipanche* lineage may have had to connect by chance to a deep branch. Thus, the convergence hypothesis is not supported. Because the breadth of *Phelipanche* and *Orobanche* species we have sampled spans the deepest branches of broomrape diversity [55], the albumin gene can be inferred to have survived through an extended evolutionary radiation of at least 150 species [55-57] or more, if the number of now-extinct broomrape species could be estimated.

Because the introns of *Phelipanche* albumin 1 xenogenes maintain critical splicing sites and share the same starting positions and first nine base pairs with the known *M. truncatula* albumin 1 intron, it is likely that the HGT event in broomrapes involved transfer of a genomic sequence rather than a separate cDNA. Following the transfer, albumin 1 genes in broomrape species have evolved under purifying selection consistent with what is observed in related legume albumin 1 genes. This observation, as well as the stage-specific transcription patterns, conserved cysteine residues and predicted 3D KNOTTIN protein structures, strongly suggest that albumin 1 genes encode functional proteins in broomrape species, and could potentially serve a function similar to its role in legumes, providing a large pool of sulfur storage and exhibiting toxicity to insect herbivores in certain legumes [29,30]. A recent report involves panicoid grass species with C3 or C4 photosynthetic pathways. Evidence was presented that nuclear genes were horizontally transferred between panicoid species and were subsequently adapted into the existing pathways with the effect of advancing the extent of C4 photosynthesis in some lineages [21]. These results indicate that HGT may promote the sharing of adaptive traits among related species. In comparison, the albumin example described here shows how a completely novel and highly specialized trait has been acquired at an ancestral stage from a distantly related donor species and maintained by the recipient lineage throughout an extended period of evolutionary history.

The albumin 1 genes in *P. aegyptiaca* are highly transcribed in most of the developmental stages we examined. Transcripts are more abundant in reproductive tissue, and lowest in the young haustorium (stage 3), which represents the earliest point in our tissue sampling where the parasite is in direct contact with the host plant. This suggests that the novel gene in *P. aegyptiaca* is probably not encoding a protein that is playing a direct role in the process of haustorial formation, and that albumin 1 expression is down-regulated as the parasite devotes energy to the essential process of establishing host vascular connections. It is also possible that the low expression in the haustorial stage could help the parasite avoid detection or minimize a negative impact on the health of the host plant during early stages of parasite contact and feeding.

Several other parasitic lineages, including members of *Cuscuta (Convolvulaceae)*, *Cassytha (Lauraceae)*, Apodanthaceae, Hydnoraceae, and the order Santalales, regularly feed upon legumes [58] and therefore might also have had opportunities to acquire albumin 1 sequences through HGT. Large transcriptome datasets are currently available for only two of these, the generalist parasite *Cuscuta pentagona (Convolvulaceae)* and for the legume specialist feeder *Pilostyles thurberi (Apodanthaceae)*[17]*.* Both of these parasites, and other species in these genera, feed widely on legumes. No homolog of albumin 1 was detected in BLAST searches of the *Pilostyles* transcriptome in the 1KP dataset [59]. However, albumin 1 sequences were detected in the same dataset and in two additional transcriptome libraries from *Cuscuta pentagona* (J. Westwood, unpublished data but publicly available through 1KP Blast database). Phylogenetic analysis nests the *Cuscuta* sequences well within Leguminosae, but on an independent branch from the broomrape sequences (Additional file 8: Figure S6), suggesting that these transcripts in *Cuscuta* represent a different HGT event into *Cuscuta* from a lineage of papilionoid legumes that was different from the source of the broomrape albumin 1 xenogene. The putative *Cuscuta* albumin 1 similarly encodes a protein predicted to have KNOTTIN structure (Knoter1d score: 33 to 35). No other albumin 1 sequences were identified elsewhere in searches of REFSEQ or publicly available plant transcriptome datasets.

## Conclusions

Because of their extensive, intimate contacts with host plant tissues, and the wide range of materials that are commonly transmitted across haustorial connections [27,28,46,60-63], parasitic plants play an important role as recipients and donors for HGT in plants [13][14,15,17,19,24]. As parasitic plants increasingly become the targets for genome-scale analyses, it should become possible to estimate the frequency and likely mechanisms of HGT events between parasites and hosts involving albumin 1 and other genes, the likelihood of more complex stepping-stone models, and how often HGT leads to long-term maintenance of new genes and novel traits.

## Methods

### Screening for HGT candidates

The assembled transcriptome of the parasite *P. aegyptiaca* was systematically screened for potential HGT candidate sequences. Immediately following an HGT event, a host-derived sequence in a parasitic organism may be identical to the sequence from the host. Over evolutionary time, the host-derived sequence will diverge from the ancestral transgene and, if it survives long enough, the xenologous sequence may pass through both speciation events (forming “xenorthologs”) and/or duplication events (forming “xenparalogs”). Initially, the xenologous sequence will be more closely related to the host sequence than to any other sequence in the parasite or its relatives’ genomes. Such sequences can provide valuable indicators of the rate and types of host-derived sequence incorporation in parasite-host interactions, but they can be difficult to distinguish from host-plant contamination or host-derived mobile transcripts in the parasite. However, as genetic divergence, speciation, and gene duplication events occur, the xenologs can be detectable as a clade of sequences that is closely related to sequences from the host lineage.

The parasitic plants that are the focus of this study are in the family Orobanchaceae (eudicots, asterid order Lamiales). The analysis begins with high throughput BLAST (tBLASTx) of all the contigs from the *P. aegyptiaca* transcriptome assembly against a database with sequences from two closely related nonparasitic species (*Lindenbergia philippensis*, a member of Orobanchaceae, representing the nonparasitic sister group of the parasitic members, and *Mimulus guttatus*, another closely related nonparasitic species of Lamiales/Asteridae, [64]) and thirteen other plant species with sequenced genomes or large transcriptome assemblies, including eudicots (two Solanaceae [asterids related to Lamiales]: *Solanum lycopersicum and Nicotiana tabacum;* and six much more distantly related rosid taxa including the range of major host families for most broomrapes: *Arabidopsis thaliana* [Brassicaceae], *Carica papaya* [Caricaceae], *Populus trichocarpa* [Salicaceae], *Medicago truncatula* [Fabaceae, papilionoid], *Cucumis sativus* [Cucurbitaceae], *Vitis vinifera* [Vitaceae]) *monocots (Sorghum bicolor, Oryza sativa)* and distantly related non-vascular plant species *(Selaginella moellendorffii, Physcomitrella patens, Chlamydomonas reinhardtii*). Details about the database are in Additional file 9: Table S3. The analysis details are described below.

Contigs were downloaded from the Parasitic Plant Genome Project website (Assembly version OrAeBC4). The HGT candidate screening includes the following steps. First, contigs were BLASTed onto the queried database (tBLASTx, expected value: 1e-10, -b 1, -v 1) described in the above paragraph and the top hit of the BLAST result was retrieved. Second, contigs with rosid species as the top hit were maintained for downstream filtering processes to identify sequences that could be useful for high-resolution evolutionary analysis. Candidate sequences were retained only if the contig length was longer than five hundred base pairs, the aligned identity score was in the range of sixty to ninety five percent, and aligned length was at least fifty percent of the contig length. The last requirement was included to avoid long contigs that only have a small portion that is nearly identical to a distantly related sequence. Third, the filtered contigs were BLASTed against the same database and the top ten hits (expected value: 1e-10, -b 10, -v 10) were retrieved. Contigs that had either of the closely related *Mimulus guttatus* or *Lindenbergia philippensis* present in the top ten hits were excluded from further consideration to avoid sequences that were not decisively better matches to distantly related species. Fourth, the same BLAST was performed for the contigs that have passed the previous screenings and all the BLAST hits (expected value: 1e-10, -b 100000, -v 100000) available were considered. If a contig had no *Mimulus guttatus* and *Lindenbergia philippensis* in the BLAST hits, which would be expected if the sequence were vertically transmitted from a nonparasitic ancestor, such a contig would be considered as a HGT candidate. However, if a contig had *Mimulus guttatus* or *Lindenbergia philippensis* among the BLAST hits, but there was much higher expect value or a much smaller bit score to a host plant lineage, such a contig was also retained as a HGT candidate. We initially began with 157806 *Phelipanche aegyptiaca* contigs. 333 contigs passed the initial BLAST screening, while 168 contigs and 36 contigs passed the second and third BLAST screenings, respectively. These 36 HGT candidates were passed on to phylogenetic testing. Once HGT candidates were found, we also checked for related sequences in the other parasitic Orobanchaceae species *Striga hermonthica* and *Triphysaria versicolor* by using BLAST search, including psi-BLAST*.*

### Phylogenetic analysis and dating

Phylogenetic analysis was performed on all albumin1 homologs detected in the broomrape species (*Phelipanche, Orobanche*) as well as all other previously known albumin1 sequences and sequences obtained from additional legume species via PCR and cloning (see below). Albumin1 is reported to be restricted to papilionoid legume species (including *Medicago*). Low stringency BLAST searches (using E-value cutoff of e-5; tBLASTx, BLASTp, and psiBLAST) of diverse angiosperm databases including NCBI nr database, PlantGDB, Phytozome database and SOL genome network (Versions of all databases are before May 2012), failed to detect any additional homologs outside legumes. MUSCLE [65] was used to produce a multiple sequence alignment of the translated amino acid sequences; a custom java program was used to force nucleotide sequences onto the corresponding amino acid alignment sequences to yield a DNA sequence alignment consistent with the translated sequences. ML phylogeny was obtained using RAxML, version 7.0.4 [66] with the following parameters: raxmlHPC –f a –x 12345 –p 12345 -# 100 –m GTRGAMMA –s alignmentsFile –n OutputFile. Multiple sequence alignments and phylogeny files were deposited in TreeBASE with submission ID: 138787 (http://purl.org/phylo/treebase/phylows/study/TB2:S13878). Genomic sequence data could be downloaded from the following link, http://www.atcgu.com/albumin1_HGT_BMC_data.zip. Bayesian analysis was performed with BEAST version 1.6.1 [67], using the following parameters: substitution model : GTR, base frequencies : estimated, site heterogeneity model : gamma, clock model : relaxed clock (uncorrelated exp), tree prior : speciation (yule process), MCMC : length of chain 10000000, Log parameters every 1000 chain. Tracer version 1.4 [67] was used to determine the performance of the BEAST output. Tracer burn-in state is 1000000. All ESS are larger than 196.

The potential HGT acquisition time was estimated by BEAST v1.6.1 using the same alignment. We assigned one calibration point: the most recent common ancestor (MRCA) of *Pisum/Medicago/Astragalus/Onobrychis*, of which the prior was treated as fitting a normal distribution with mean set to 39 mya and stdev of 2.4 mya [51]. We also created taxon groups of *Onobrychis/Orobanche/Phelipanche*, *Orobanche/Phelipanche*, and a taxon group just containing *Phelipanche* genes. The other settings are the same as described above in Phylogenetic analysis section. Tracer was used to analyze the output of BEAST to report the estimated mean and 95% HPD range of divergence time of the previously defined taxon groups (16 Mya: 95% HPD is 11-21 mya. 11 Mya: 95% HPD is 6-16 Mya. 5 Mya: 95% HPD is 3-7 my.). Similar patterns were observed within the BEAST confidence ranges when dates were estimated with r8s [68] (results not shown).

### KNOTTIN structure validation and 3D structure simulation

HGT candidates were confirmed to be KNOTTIN proteins using the prediction program provided by the KNOTTIN database [31,69]. Amino acid sequences were first confirmed as KNOTTIN structures using Knoter1D program offered by the KNOTTIN database. Knoter1D scores larger than 20 are determined to be KNOTTIN protein structures. Confirmed amino acid sequences (all the albumin1 sequences in *Phelipanche*) were input in Knoter1D3D program and pdb files were generated by this program.

### dN, dS and dN/dS calculation

HyPhy version 2.0 was used to calculate dN, dS and dN/dS ratios [70]. Treefiles and multiple sequence alignments of albumin 1 coding sequences were imported into HyPhy with the ML phylogeny based on the above analysis. Analyses were focused on broomrape species plus three most closely related legume species. Calculations were performed using the following parameters: partition type: codon; substitution model: MG94xHKY85_3x4; parameters: local; equilibrium freqs: estimate. HyPhy was also used in functional constraint analyses among sites using the empirical Bayes technique, detailed results are in Additional file 10: Table S4.

### Expression level comparisons of HGT candidates

Assembled contigs and raw Illumina reads were downloaded from PPGP website. For each library, raw reads were mapped onto the HGT candidates in *P. aegyptiaca* using bwa [71], samtools [72] and bedtools [73]. Normalized measures of expression intensity, Reads Per Kilobase per Million mapped reads (RPKM), were calculated from the read counts, the length of each contig, and the total number of mapped reads in each library or developmental stage [54].

### Obtaining genomic sequences by PCR approach

#### Broomrape species DNA extraction, and gene amplification

Two different sources of tissue were used for broomrape species, dry seeds (obtained from the GermPlasm Bank of the IAS-CSIC, Cordoba, Spain) for *Orobanche ballotae, Orobanche hederae, Phelipanche nana* and *Phelipanche schultzii*, and vegetative shoots for *Phelipanche aegyptiaca*, *Orobanche cernua, Orobanche minor, Phelipanche mutelii*, and *Phelipanche ramosa*. Total genomic DNA was isolated from fresh, liquid nitrogen frozen tissue using a DNeasy Plant Mini Kit (Qiagen).

EST unigene contigs OrAeGnB1_75797 and OrAe41G2B1_12653 were downloaded from the Parasitic Plant Genome Project database. A different set of *P. aegyptiaca* specific primers was designed for each contig (Additional file 11: Table S5). The *P. aegyptiaca* primers were also used to amplify related sequences from other *Orobanche* species *P. mutelii, P. nana, P. ramosa* and *P. schultzii*. Each PCR reaction contained 10 ng of genomic DNA, 0.5 μM of each forward and reverse primers, 12.5 of 2x iProof Master Mix (BIO-RAD) and conditions as described in the manufacturer’s protocol. PCR products were separated by electrophoresis through a 1% agarose gel, yielding a single band that was excised from the gel, purified using the QIAquick Gel extraction kit (Qiagen), and sequenced using ABI3730xl genetic analyzer and Big Dye Terminator v3.1 sequencing kit for sequencing (both from Applied Biosystems).

### Legume DNA extraction, and gene amplification

Total DNA was isolated from herbarium material of *Onobrychis argentea* Boiss. ssp. africana, A. Dubois 13246 (M), using a DNeasy Plant Mini Kit (Qiagen). Because the *Onobrychis* sequence obtained from NCBI was incomplete, one forward primer (AlbuminFw3: ^5´^TTAAGCTCACTCCTTTGGTCCTCTTC^3´^) and one degenerate reverse primer (AlbuminRv3: ^5´^CAGGCATCTTCARGAAKCYTTTYKC^3´^) were designed in order to amplify the full length Albumin 1 gene in *O. argentea*. Forward 3 was designed on the Q6A1C9 sequence, targeting the more conserved region before the start codon between sequences Q6A1C9 and Q6A1D7 obtained from *Onobrychis viciifolia* and *Astragalus monspessulanus.* Reverse 3 was designed from the downstream end of the complete albumin genes Medtr7g041000.1 and OrAeGnB1_75797. The PCR reaction was composed by 10 ng of genomic DNA of *O. argentea*, using forward primer (Fw3, 1 μM), reverse primer (Rv3, 1 μM), and 12.5 μl of 2x iProof Master Mix (BIO-RAD) in a final volume of 25 μl., following the manufacturer’s protocol. PCR product was separated by electrophoresis through a 1% agarose gel. This product was excised from the gel, purified using the QIAquick Gel extraction kit (Qiagen), sequenced and identified as Albumin 1.

## Competing interests

The authors declare that they have no competing interests.

## Authors’ contributions

Conception and design of PPGP transcriptome study (JHW, CWD, MPT, JIY); conception and design of HGT study (YZ, CWD); *Phelipanche* and *Orobanche* plants, DNAs, PCR, cloning, and chromosome walking (MF-A, JHW); plants, RNAs, and libraries for transcriptome sequencing (MF-A; LAH, PER, MD), legume DNAs (MFW), data analysis and presentation (YZ, EKW, YJ, NJW, MF-A, CWD); wrote manuscript (YZ and CWD, with contributions from all of the authors). All authors read and approved the final manuscript.

## Supplementary Material

Additional file 1: Figure S1 Phylogeny of major lineage of plants, adapted from Soltis et al [42]. Legumes belong to the rosid order Fabales (blue box), while the parasites *Phelipanche* and *Cuscuta* represent derived lineages within the asterid orders Lamiales, (red box) and Solanales (green box), respectively. Click here for file

Additional file 2: Figure S2 NCBI BLAST result (database: nr, BLASTp) of (A) *P. aegyptiaca* albumin1-1 (unigene 12653) and (B) *P. aegyptiaca* albumin1-2 (unigene 75797). Click here for file

Additional file 3: Figure S3 Amino acid alignment of insect toxin albumin 1 protein (*Medicago_truncatula*_albumin1_Q7XZC5) and inferred protein sequences for the two homologs in *P. aegyptiaca*, and (B) structure of the *M. truncatula* toxic albumin 1 gene. **(A)** Inferred protein sequence alignments are 57.3-58.3% identical and 72.7%-74.3% similar (= identity + conservative substitutions) in shared regions between the legume and parasite proteins. **(B)** The legume protein product has a 27 amino acid signal peptide and 113 amino acid mature peptide; both regions are similarly conserved between the legume and *Phelipanche* inferred proteins. The gene structure representation for this legume gene was obtained from EMBL-EBI databases [74] (accession #AJ574789). Click here for file

Additional file 4: Figure S4 Alignments of the 3’ end of genomic and inferred CDS sequences of albumin 1 homologs from five *Phelipanche* species. Two genes are identified from *P. aegyptiaca* unigene 12653 (first five sequences, red bar) and unigene 75797 (yellow bar)*.* Red box indicates putative stop codon. Click here for file

Additional file 5: Figure S5 Partial genomic DNA and cDNA alignments of *M. truncatula* albumin 1 (Medtr8g025950), *P. aegyptiaca* albumin1-1 (12653) and *P. aegyptiaca* albumin 1-2 (75797). Intron start and end positions are illustrated by arrows. Click here for file

Additional file 6: Table S1 Expression values for albumin 1 genes in *P. aegyptiaca* at different developmental stages. Expression levels were measured by number of mapped Reads to this gene Per Kilobase of sequence length per Million (M) library reads (RPKM) in Illumina sequence (G) libraries (PPGP). Developmental stages described in Table S2. Click here for file

Additional file 7: Table S2 Developmental stages used for transcriptome sequencing in *P. aegyptiaca*[54] with characteristics of each stage and the expectation of host plant tissue contamination in library preparations. Click here for file

Additional file 8: Figure S6 Maximum likelihood (ML) phylogeny of KNOTTIN homologs in broomrape species, *Cuscuta pentagona* and papilionoid legumes. ML and Bayesian Inference (BI) methods produced the same tree topology. Three *Cuscuta pentagona* sequences were obtained from the 1KP project and from additional independently prepared libraries. Other information as given (Figure 2). Click here for file

Additional file 9: Table S3 HGT candidates BLAST database. Information that cannot be retrieved is marked as Not Applicable (NA). M: million; GB: Gigabase. Click here for file

Additional file 10: Table S4 Evolutionary constraints in albumin 1 genes in *Phelipanche* and related legumes. Click here for file

Additional file 11: Table S5 PCR primers used for albumin 1 amplification. Click here for file
