# Supplementary figures and images for: Evolution of a horizontally acquired legume gene, albumin 1, in the parasitic plant Phelipanche aegyptiaca and related species
Source: BMC Evol Biol. 2013 Feb 20;13:48. doi: 10.1186/1471-2148-13-48 (PMC3601976; doi:10.1186/1471-2148-13-48)

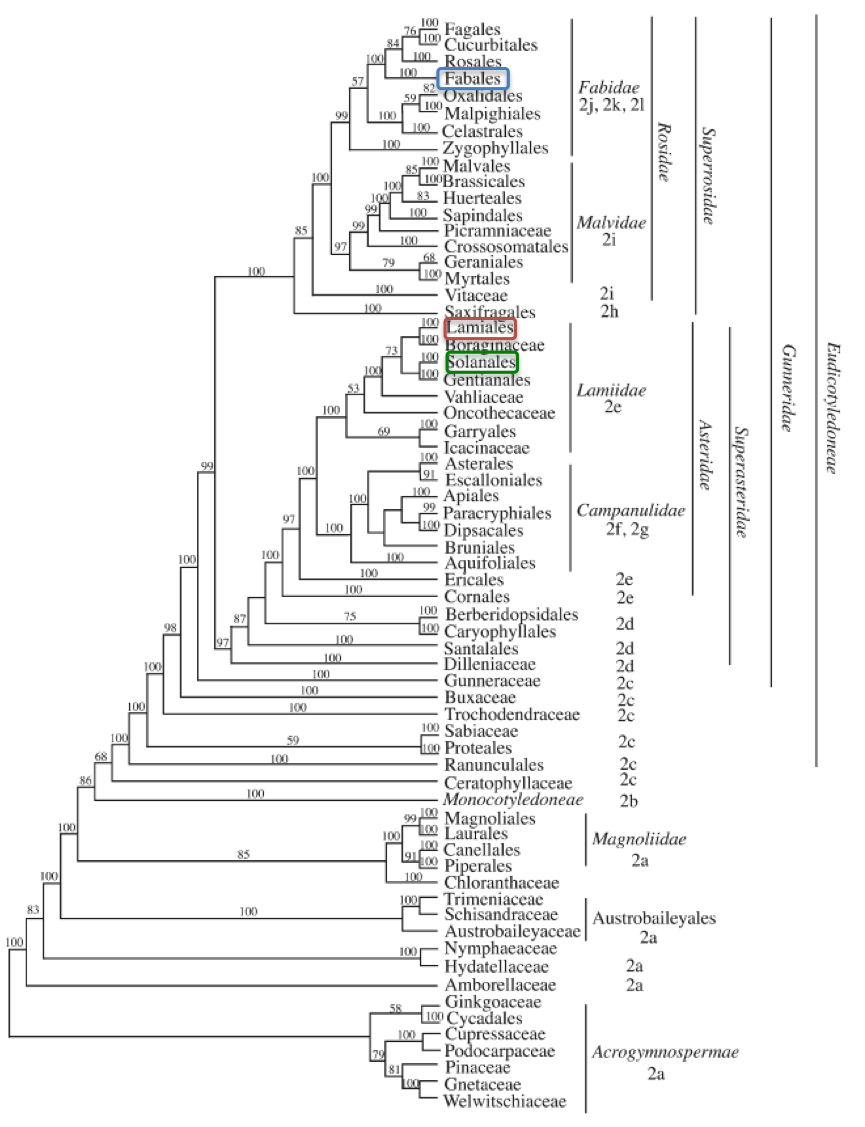

Supplement: Additional file 1: Figure S1 — Phylogeny of major lineage of plants, adapted from Soltis et al [42]. Legumes belong to the rosid order Fabales (blue box), while the parasites Phelipanche and Cuscuta represent derived lineages within the asterid orders Lamiales, (red box) and Solanales (green box), respectively. [file 1471-2148-13-48-S1.png]

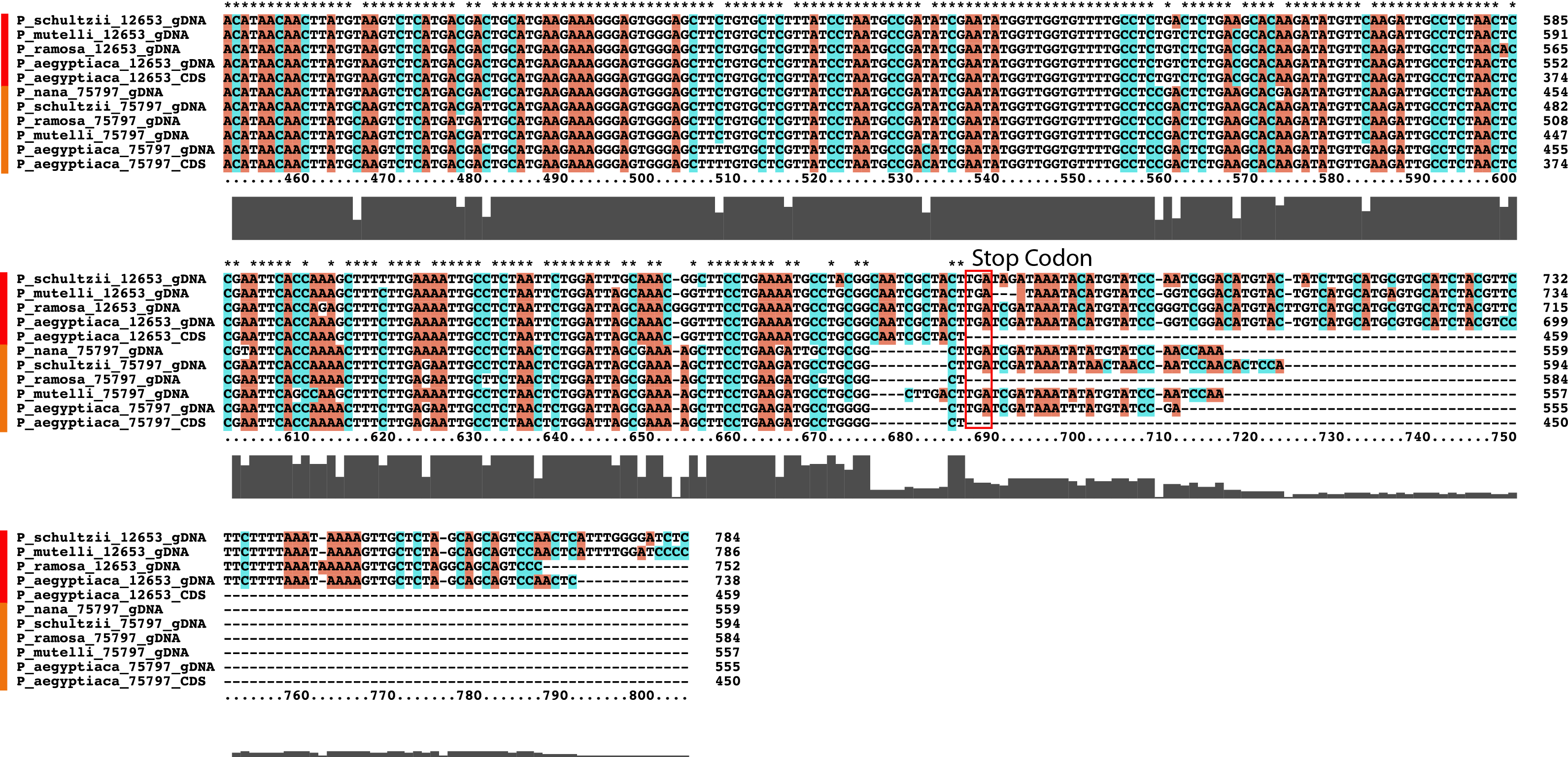

Supplement: Additional file 4: Figure S4 — Alignments of the 3’ end of genomic and inferred CDS sequences of albumin 1 homologs from five Phelipanche species. Two genes are identified from P. aegyptiaca unigene 12653 (first five sequences, red bar) and unigene 75797 (yellow bar). Red box indicates putative stop codon. [file 1471-2148-13-48-S4.png]

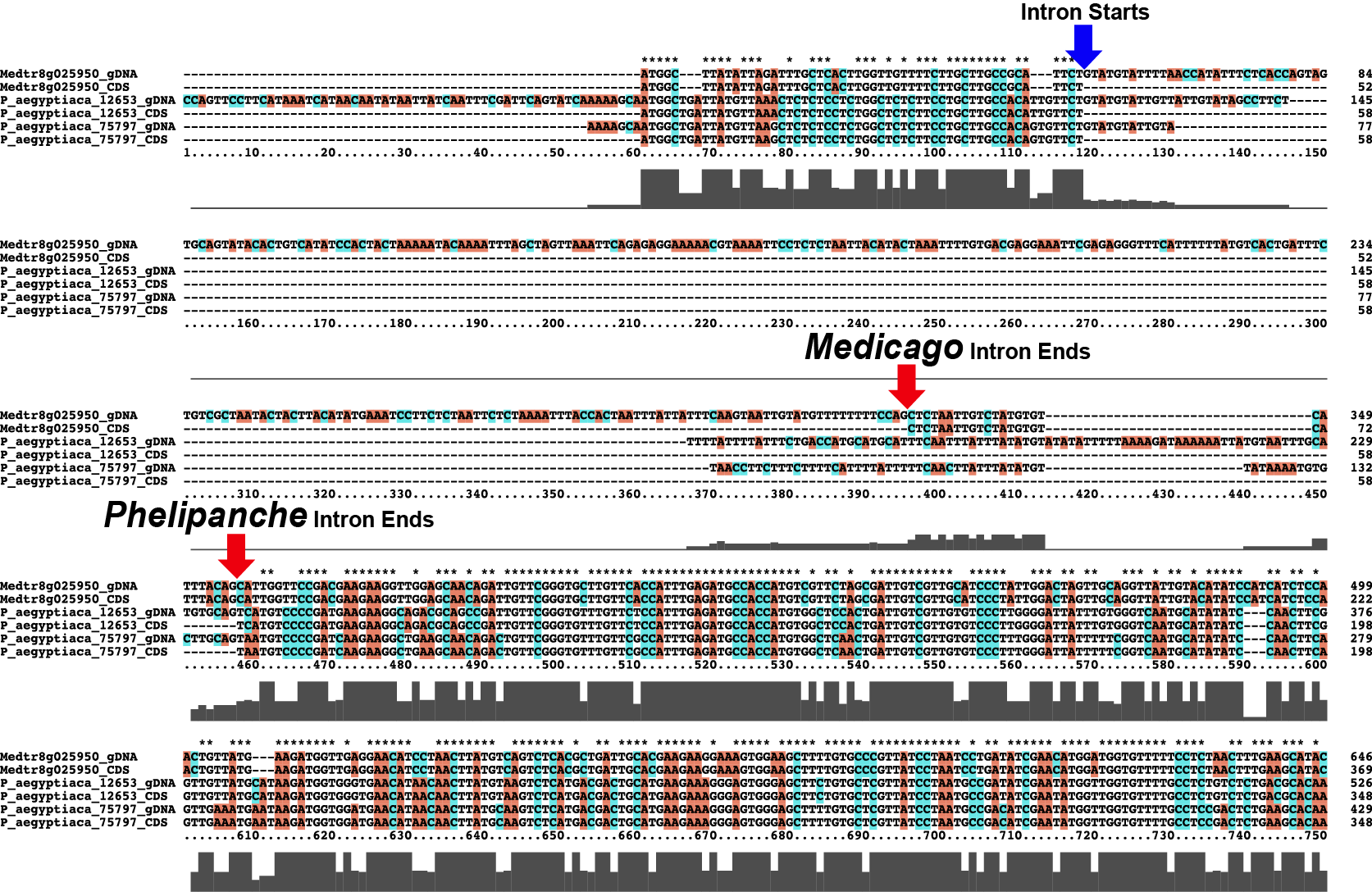

Supplement: Additional file 5: Figure S5 — Partial genomic DNA and cDNA alignments of M. truncatula albumin 1 (Medtr8g025950), P. aegyptiaca albumin1-1 (12653) and P. aegyptiaca albumin 1-2 (75797). Intron start and end positions are illustrated by arrows. [file 1471-2148-13-48-S5.png]

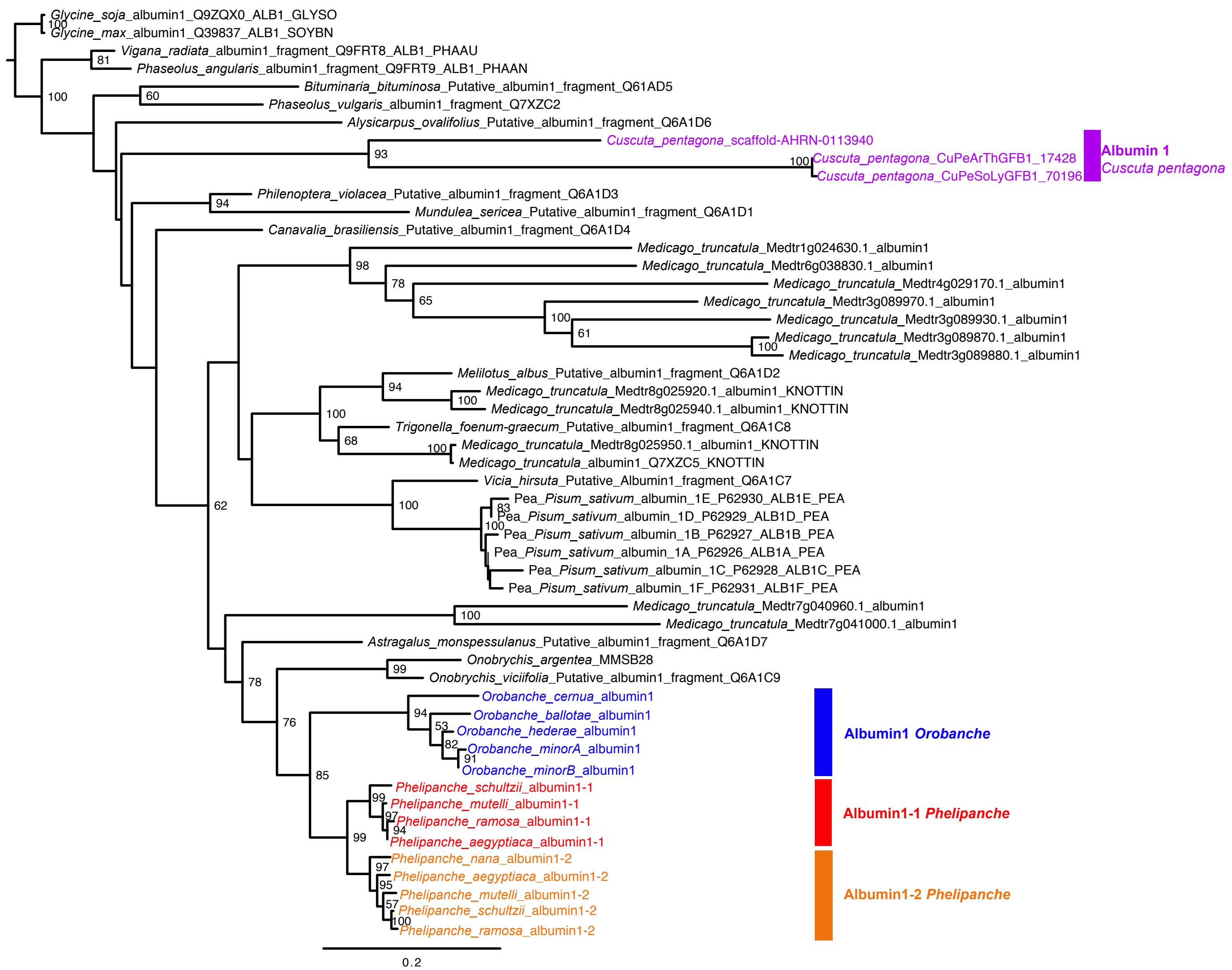

Supplement: Additional file 8: Figure S6 — Maximum likelihood (ML) phylogeny of KNOTTIN homologs in broomrape species, Cuscuta pentagona and papilionoid legumes. ML and Bayesian Inference (BI) methods produced the same tree topology. Three Cuscuta pentagona sequences were obtained from the 1KP project and from additional independently prepared libraries. Other information as given (Figure 2). [file 1471-2148-13-48-S8.pdf]
