# Supplementary material for: Evolution of a horizontally acquired legume gene, albumin 1, in the parasitic plant Phelipanche aegyptiaca and related species
Source: BMC Evol Biol. 2013 Feb 20;13:48. doi: 10.1186/1471-2148-13-48 (PMC3601976; doi:10.1186/1471-2148-13-48)
Supplement: Additional file 2: Figure S2 — NCBI BLAST result (database: nr, BLASTp) of (A) P. aegyptiaca albumin1-1 (unigene 12653) and (B) P. aegyptiaca albumin1-2 (unigene 75797). [file 1471-2148-13-48-S2.pdf]

A

```
>emb|CAE00461.1|  albumin 1 [Medicago truncatula]
Length=140

 Score =  166 bits (419),  Expect = 5e-51, Method: Compositional matrix adjust.
 Identities = 81/144 (56%), Positives = 99/144 (69%), Gaps = 13/144 (9%)

Query  4    YVKLSPLALFLLATVFLMSPIKKAEATDCSGVCSPFEMPPCGSTDCRCVPLGLFFGQCIY  63
            Y++ + L +FLLA  F + P KK  ATDCSG CSPFEMPPC S+DCRC+P+GL  G C Y
Sbjct  3    YIRFAHLVVFLLA-AFSLVPTKKVGATDCSGACSPFEMPPCRSSDCRCIPIGLVAGYCTY  61

Query  64   PTSVEMNKMVDEHNNLCKSHDDCMKKGSGSFCARYPNADIEYGWCFASDSEAQDMLKIAS  123
            P+S   + KMV+EH NLC+SH DC KK SGSFCARYPN DIE+GWCF+S+ EA D+
Sbjct  62   PSSPTVMKMVEEHPNLCQSHADCTKKESGSFCARYPNPDIEHGWCFSSNFEAYDV-----  116

Query  124  NSEFTKTFLRIASNSGLAKSFLKM  147
                   F  ++SN GL K  L M
Sbjct  117  -------FFNVSSNRGLIKDSLPM  133
```

B

```
>ref|XP_003627383.1|  Albumin [Medicago truncatula]
 gb|AET01859.1|  Albumin [Medicago truncatula]
Length=145

 GENE ID: 11420704 MTR_8g022400 | Albumin [Medicago truncatula]

 Score =  160 bits (404),  Expect = 1e-48, Method: Compositional matrix adjust.
 Identities = 77/127 (61%), Positives = 96/127 (76%), Gaps = 2/127 (2%)

Query  4    YVKLSPLALFLLATLFFMSPMKKADAADCSGVCSPFEMPPCGSTDCRCVPWGLFVGQCIY  63
            Y++L+ L +FLLAT   + PM KA A DC G+CSPFEMPPC S+ CRC+P  L  G C+
Sbjct  3    YLRLAHLVVFLLATFSLIFPMMKA-AEDCLGICSPFEMPPCPSSSCRCIPVILIGGNCVD  61

Query  64   PTSVVMHKMVGEHNNLCKSHDDCMKKGSGSFCARYPNADIEYGWCFASVSDAQDM-FKIA  122
            P+S   + KMV +H NLC+SH DC KKGSGSFCARYPN DIEYGWCF+S S AQ++ F+I+
Sbjct  62   PSSPTITKMVEKHANLCQSHADCTKKGSGSFCARYPNPDIEYGWCFSSSSRAQEVFFEIS  121

Query  123  SNSEFTK  129
            SN +F K
Sbjct  122  SNPQFIK  128
```
