# Supplementary material for: Evolution of a horizontally acquired legume gene, albumin 1, in the parasitic plant Phelipanche aegyptiaca and related species
Source: BMC Evol Biol. 2013 Feb 20;13:48. doi: 10.1186/1471-2148-13-48 (PMC3601976; doi:10.1186/1471-2148-13-48)
Supplement: Additional file 3: Figure S3 — Amino acid alignment of insect toxin albumin 1 protein (Medicago_truncatula_albumin1_Q7XZC5) and inferred protein sequences for the two homologs in P. aegyptiaca, and (B) structure of the M. truncatula toxic albumin 1 gene. (A) Inferred protein sequence alignments are 57.3-58.3% identical and 72.7%-74.3% similar (= identity + conservative substitutions) in shared regions between the legume and parasite proteins. (B) The legume protein product has a 27 amino acid signal peptide and 113 amino acid mature peptide; both regions are similarly conserved between the legume and Phelipanche inferred proteins. The gene structure representation for this legume gene was obtained from EMBL-EBI databases [74] (accession #AJ574789). [file 1471-2148-13-48-S3.pdf]

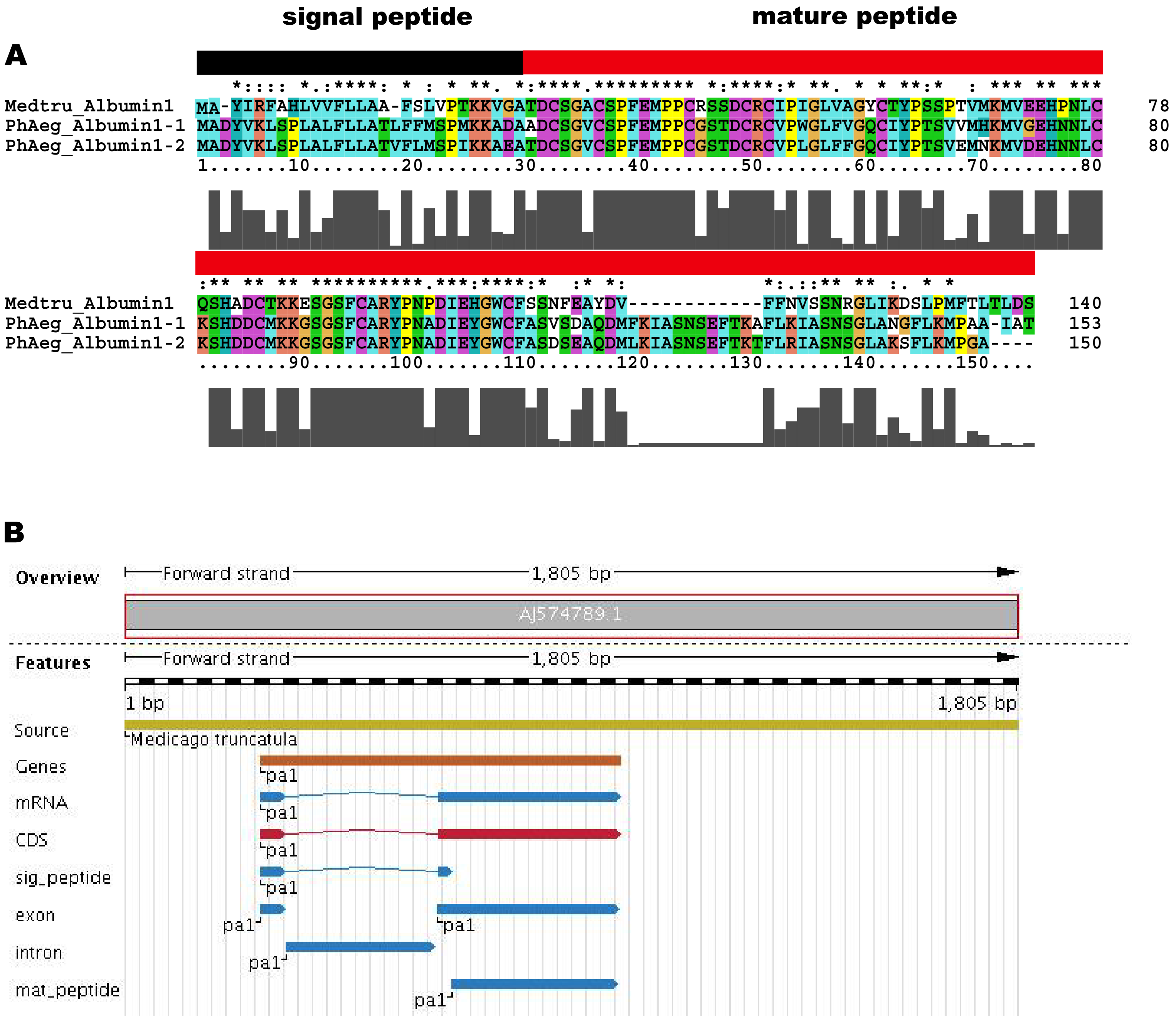
A
signal peptide
mature peptide
Medtru_Albumin1 MA-YIRFAHLVVFLLAA-FSLVPTKKVGATDCSGACSPFEMPPCRSSDCRCIPIGLVAGYCTYPSSPTVMKMVEEHPNLC 78
PhAeg_Albumin1-1 MADYVKLSPLALFLLATLFFMSPMKKADAADCSGVCSPFEMPPCGSTDCRCVPWGLFVGQCIYPTSVVMHKMVGEHNNLC 80
PhAeg_Albumin1-2 MADYVKLSPLALFLLATVFLMSPIKKAEATDCSGVCSPFEMPPCGSTDCRCVPLGLFFGQCIYPTSVEMNKMVDEHNNLC 80
Medtru_Albumin1 QSHADCTKKESGSFCARYPNPDIEHGWCFSSNFEAYDV-----------FFNVSSNRGLIKDSLPMFTLTLDS 140
PhAeg_Albumin1-1 KSHDDCMKKGSGSFCARYPNADIEYGWCFASVSDAQDMFKIASNSEFTKAFLKIASNSGLANGFLKMPAA-IAT 153
PhAeg_Albumin1-2 KSHDDCMKKGSGSFCARYPNADIEYGWCFASDSEAQDMLKIASNSEFTKTFLRIASNSGLAKSFLKMPGA---- 150
B
Overview
Forward strand
1,805 bp
AJ574789.1
Features
Forward strand
1,805 bp
1 bp
1,805 bp
Source
Medicago truncatula
Genes
pa1
mRNA
pa1
CDS
pa1
sig_peptide
pa1
exon
pa1
pa1
intron
pa1
mat_peptide
pa1
