# Supplementary material for: Evolution of a horizontally acquired legume gene, albumin 1, in the parasitic plant Phelipanche aegyptiaca and related species
Source: BMC Evol Biol. 2013 Feb 20;13:48. doi: 10.1186/1471-2148-13-48 (PMC3601976; doi:10.1186/1471-2148-13-48)
Supplement: Additional file 6: Table S1 — Expression values for albumin 1 genes in P. aegyptiaca at different developmental stages. Expression levels were measured by number of mapped Reads to this gene Per Kilobase of sequence length per Million (M) library reads (RPKM) in Illumina sequence (G) libraries (PPGP). Developmental stages described in Table S2. [file 1471-2148-13-48-S6.docx]

**Table S1**. Expression values for albumin 1 genes in *P. aegyptiaca* at different developmental stages. Expression levels were measured by number of mapped Reads to this gene Per Kilobase of sequence length per Million (M) library reads (RPKM) in Illumina sequence (G) libraries (PPGP). Developmental stages described in **Table S2**.

| Albumin I genes | 0G | 1G | 2G | 3G | 41G | 42G | 61G | 62G |
| --- | --- | --- | --- | --- | --- | --- | --- | --- |
| *Albumin1-1* | 96.5 | 71 | 186.2 | 22.6 | 78 | 29 | 414 | 9402.3 |
| *Albumin1-2* | 186.3 | 237.1 | 368.4 | 6.5 | 169.5 | 40.9 | 2826.5 | 23162.7 |
| Total reads used in mapping (M) | 15.6 | 26 | 25.9 | 18.2 | 27.9 | 20.7 | 15.8 | 16.2 |
