# Supplementary material for: Evolution of a horizontally acquired legume gene, albumin 1, in the parasitic plant Phelipanche aegyptiaca and related species
Source: BMC Evol Biol. 2013 Feb 20;13:48. doi: 10.1186/1471-2148-13-48 (PMC3601976; doi:10.1186/1471-2148-13-48)
Supplement: Additional file 7: Table S2 — Developmental stages used for transcriptome sequencing in P. aegyptiaca[54] with characteristics of each stage and the expectation of host plant tissue contamination in library preparations. [file 1471-2148-13-48-S7.docx]

**Table S2**. Developmental stages used for transcriptome sequencing in *P. aegyptiaca* [72] with characteristics of each stage and the expectation of host plant tissue contamination in library preparations.

| Stage Name | Expectation of Host Plant Tissues Contamination | Description of the developmental stage | More Information |
| --- | --- | --- | --- |
| 0 | No | Seeds imbibed, pre-germination | Pre-attachment of haustoria |
| 1 | No | Germinated seed; Radicle emerged; pre-haustorial growth |  |
| 2 | No | Seedling after exposure to haustorial induction factors (HIFs) |  |
| 3 | Yes | Haustoria attached to host root; early penetration stages, pre-vascular connection (~48 hrs.) | Early post-attachment |
| 4.1 | Yes | Early established parasite; parasite vegetative growth after vascular connection (~72 hrs.) |  |
| 4.2 | Yes | Spider stage |  |
| 5.1 | No | Pre-emergence from soil - shoots | Late post-attachment |
| 5.2 | No | Pre-emergence from soil - roots |  |
| 6.1 | No | Post emergence from soil - Vegetative structures; leaves/stems |  |
| 6.2 | No | Post emergence from soil - Reproductive structures; floral buds (up to anthesis) |  |
