# Supplementary material for: Evolution of a horizontally acquired legume gene, albumin 1, in the parasitic plant Phelipanche aegyptiaca and related species
Source: BMC Evol Biol. 2013 Feb 20;13:48. doi: 10.1186/1471-2148-13-48 (PMC3601976; doi:10.1186/1471-2148-13-48)
Supplement: Additional file 9: Table S3 — HGT candidates BLAST database. Information that cannot be retrieved is marked as Not Applicable (NA). M: million; GB: Gigabase. [file 1471-2148-13-48-S9.docx]

**Table S3**. HGT candidates BLAST database. Information that cannot be retrieved is marked as Not Applicable (NA). M: million; GB: Gigabase.

|  |  | Classification | Resource | *# of Reads* | *Size of*  *Dataset* | *# of Unigenes* |
| --- | --- | --- | --- | --- | --- | --- |
| Closely related species | *Striga hermonthica* | Eudicots,Asterids,Lamiids,  Lamiales,Orobanchaceae | PPGP | 473.3M | 41GB | 726534 |
|  | *Triphysaria versicolor* | Eudicots,Asterids,Lamiids,  Lamiales,Orobanchaceae | PPGP | 181M | 15.5GB | 480595 |
|  | *Lindenbergia philippensis* | Eudicots,Asterids,Lamiids,  Lamiales,Orobanchaceae | PPGP | 69M | 5.9GB | 104904 |
|  | *Mimulus guttatus* | Eudicots,Asterids,Lamiids,  Lamiales,Phrymaceae | Phytozome | NA | NA | 27501 |
| Other more distantly related plant species | *Solanum lycopersicum* | Eudicots,Asterids,Solanales,  Solanaceae | PlantGDB | NA | NA | 56845 |
|  | *Nicotiana tabacum* | Eudicots,Asterids,Solanales,  Solanaceae | PlantGDB | NA | NA | 131942 |
|  | *Arabidopsis thaliana* | Eudicots,Rosids,Brassicales,  Brassicaceae | TAIR 9 | NA | NA | 27379 |
|  | *Carica papaya* | Eudicots,Rosids,Brassicales,  Caricaceae | ASGPB release | NA | NA | 25536 |
|  | *Populus trichocarpa* | Eudicots,Rosids,Malpighiales,  Salicaceae | JGI version 2.0 | NA | NA | 41377 |
|  | *Medicago truncatula* | Eudicots,Rosids,Fabales,Fabaceae | Phytozome | NA | NA | 50962 |
|  | *Cucumis sativus* | Eudicots,Rosids,Cucurbitales,  Cucurbitaceae | BGI release | NA | NA | 21635 |
|  | *Vitis vinifera* | Eudicots,Rosids,Vitales,Vitaceae | Genoscope release | NA | NA | 30434 |
|  | *Sorghum bicolor* | Monocots,Poales,Poaceae | JGI version 1.4 | NA | NA | 34496 |
|  | *Oryza sativa* | Monocots,Poales,Poaceae | RGAP release 6.1 | NA | NA | 56979 |
|  | *Selaginella moellendorffii* | Embryophyta,Tracheophyta,  Lycopodiophyta,Isoetopsida,  Selaginellales,Selaginellaceae | JGI version 1.4 | NA | NA | 34697 |
|  | *Physcomitrella patens* | Bryophyta,Bryophytina,  Bryopsida,Funariidae,  Funariales,Funariaceae | JGI version 1.1 | NA | NA | 35938 |
|  | *Chlamydomonas reinhardtii* | Chlorophyta,Chlorophyceae,  Chlamydomonadales,  Chlamydomonadaceae | Phytozome | NA | NA | 15935 |
