# Supplementary material for: Evolution of a horizontally acquired legume gene, albumin 1, in the parasitic plant Phelipanche aegyptiaca and related species
Source: BMC Evol Biol. 2013 Feb 20;13:48. doi: 10.1186/1471-2148-13-48 (PMC3601976; doi:10.1186/1471-2148-13-48)
Supplement: Additional file 10: Table S4 — Evolutionary constraints in albumin 1 genes in Phelipanche and related legumes. [file 1471-2148-13-48-S10.docx]

**Table S4**. Evolutionary constraints in albumin 1 genes in *Phelipanche* and related legumes.

| A. Point estimates of nonsynonymous (dN) and synonymous (dS) rates, and the dN/dS ratio: |  |  |  |
| --- | --- | --- | --- |
| Albumin 1 | dN | dS | dN/dS |
| *Astragalus monspessulanus* | 0.259 | 0.629 | 0.412 |
| *Onobrychis viciifolia* | 0 | 0.263 | 0 |
| *Onobrychis argentea* | 0.112 | 0.450 | 0.249 |
| *Orobanche* | 0.123 | 1.068 | 0.115 |
| *Phelipanche spp. Albumin1-1* | 0.043 | 0.264 | 0.163 |
| *Phelipanche spp. Albumin1-2* | 0.018 | 0.079 | 0.228 |
|  |  |  |  |
|  |  |  |  |
| B. 95% Confidence interval estimates of non-synonymous and synonymous rates: | | | |
| Albumin1 | Lower Bound | Mean | Upper Bound |
|  |  |  |  |
| *Astragalus monspessulanus* dN | 0.258439 | 0.258556 | 0.258674 |
| *Astragalus monspessulanus* dS | 0.62915 | 0.629267 | 0.629385 |
| *Onobrychis viciifolia* dN | 0 | 0 | 0.000117608 |
| *Onobrychis viciifolia* dS | 0.26275 | 0.262868 | 0.262985 |
| *Onobrychis argentea* dN | 0.112001 | 0.112119 | 0.112236 |
| *Onobrychis argentea* dS | 0.45012 | 0.450238 | 0.450355 |
| *Orobanche* dN | 0.122876 | 0.122993 | 0.123111 |
| *Orobanche* dS | 1.06818 | 1.0683 | 1.06842 |
| *Phelipanche spp. Albumin1-1* dN | 0.0428445 | 0.0429621 | 0.0430797 |
| *Phelipanche spp. Albumin1-1* dS | 0.263748 | 0.263866 | 0.263983 |
| *Phelipanche spp. Albumin1-2* dN | 0.0182241 | 0.0183417 | 0.0184593 |
| *Phelipanche spp. Albumin1-2* dS | 0.0788147 | 0.0789323 | 0.07905 |
