# Supplementary material for: Evolution of a horizontally acquired legume gene, albumin 1, in the parasitic plant Phelipanche aegyptiaca and related species
Source: BMC Evol Biol. 2013 Feb 20;13:48. doi: 10.1186/1471-2148-13-48 (PMC3601976; doi:10.1186/1471-2148-13-48)
Supplement: Additional file 11: Table S5 — PCR primers used for albumin 1 amplification. [file 1471-2148-13-48-S11.docx]

**Table S5**. PCR primers used for albumin 1 amplification.

| Primer used in | Primer | Orientation | 5’-3’sequence |
| --- | --- | --- | --- |
| Broomrape species | OrAeGnB1_75797 | Fw1 | GATTCAGCATCAAAAGCAATGGC |
|  |  | Rv1 | GGAGTGTTGGATCGGATACAT |
|  | OrAe41G2B1_12653 | Fw2 | CAACAGCAAGAACCAGTTCC |
|  |  | Rv2 | GAGATCCAACTGAGTTGGAC |
| Legumes | LegAlb1 | Fw3 | TTAAGCTCACTCCTTTGGTCCTCTTC |
|  |  | Rv3 | CAGGCATCTTCARGAAKCYTTTYKC |
